# A critical review on biogas production from edible and non-edible oil cakes

**DOI:** 10.1007/s13399-021-01292-5

**Published:** 2021-01-26

**Authors:** Anee Mohanty, Prangya Ranjan Rout, Bipro Dubey, Sumer Singh Meena, Parimal Pal, Mukesh Goel

**Affiliations:** 1 Department of Biotechnology, Dr. B. R. Ambedkar National Institute of Technology Jalandhar, Jalandhar, Punjab India; 2 Department of Biology, Hong Kong Baptist University, Kowloon Tong, Hong Kong; 3 Department of Engineering and Mathematics, Sheffield Hallam University, Sheffield, S11WB UK; 4 Department of Chemical Engineering, National Institute of Technology Durgapur, Durgapur, India

**Keywords:** Agri-waste, Edible oil cakes, Non-edible oil cakes, Biogas, Review

## Abstract

The circular economy is at the core of sustainable development. The generation of biogas from the massive quantity of agricultural waste biomass is one of the critical drivers of the circular economy. Biogas has enormous renewable energy potential and has multitudes of applications in today’s energy-intensive society. Oil cakes, a known Agri-waste, are the by-product of oil processing, and are rich in nutrients. The edible oil cakes mostly have been used as a cattle feed; however, non-edible oil cakes do not find many applications. Their production is continuously escalating as non-edible oils are increasingly used in biodiesel production. Recently, there is a lot of emphasis on biogas production from these oil cakes. This paper reviews in detail biogas production from both edible and non-edible oil cakes. Chemical composition and various other applications of the cakes are also reviewed in brief. The survey illustrates that multiple parameters such as inoculum sources, co-digestion and reactor design affect the biogas production. All those factors, along with biogas upgrading and the economy of the process, are reviewed. Finally, future research opportunities are suggested to improve the viability of the biogas production from oil cakes.

## Introduction

Population explosion, coupled with economic development, has put a serious strain on natural resources. Arguably, the most widely debated topic of the twenty-first century is how unsustainable development has resulted in increased toxic pollutants, devastating extreme climatic events, loss of biodiversity and so on. To counter the negative impacts of climate change, global efforts have been in the direction of introducing sustainable practices across all sectors. The sustainable development goals (SDGs) of the United Nations (UN) aim to align economic development with environmental protection as anthropogenic degradation of the ecosphere is a significant challenge faced by global communities today. Environmental degradation is multifaceted but at the centre lies the energy intensive economic activities. The steep rise in gross domestic product (GDP) per capita is intertwined with higher per capita energy expenditure. Ever increasing energy demand combined with ecological degradation is prompting efforts to move from fossil fuels to renewable energy sources like solar, wind, geothermal and biomass. The renewable energy potential of biomass is very high considering its easy availability; combined with technological advancements for efficient conversion of biomass to energy, it could contribute significantly to the world’s energy demand [1]. Biomass represents all the organic matter in the biosphere, and the biodegradable portion of the solid waste is the major sources of biomass that could be utilised for energy needs. Biomass generated from these sources can be directly burnt to produce energy or processed using various conversion techniques to produce biofuels like bioethanol, biodiesel and biogas. Although biomass holds enormous potential for meeting the energy needs, the current market share in the energy sector is very small [2]. Commercial biomass energy products include the first generation bioethanol production from food crops like corn, sugarcane, biodiesel from plant sources and burning of woodchips or pellets for heat/electricity, but these technologies suffer from the drawbacks like diverting food crops for the production of biofuel and the low calorific values of wood pellets.

The biggest factor contributing to environmental degradation is the sub-optimal recycling and reuse strategies [3, 4]. The success of the ambitious targets of SDGs related to clean environment and poverty elimination calls for a revamped holistic waste management strategy wherein nutrients and energy can be recovered from waste. In this regard, few bottlenecks exist in achieving the full energy potential of biomass resources addressing which can make biomass energy a significant shareholder in the world energy market. Exploring novel feedstocks for bioenergy production would contribute immensely to the biomass energy sector. Furthermore, newer technologies for the efficient conversion of lignocellulosic biomass like agricultural residues in an economically viable and environmentally sustainable way need to be explored.

## Agricultural wastes for bioenergy

The utilisation of agricultural waste biomass as feedstock for bioenergy production is primarily a lucrative option for developing nations which are majorly agrarian economies [5]. In countries like India, agro-residues are seen as a burden or waste product which are either burnt or dumped in wastelands [6]. This practice negatively impacts the environment and helps in increasing greenhouse gases (GHG) or become a breeding field for various pathogenic microorganisms.

Agricultural wastes include husk, bagasse, fruit seeds, bran, paddy straws and oil cakes generated during various stages of harvesting and processing of cereals, pulses and oilseeds. These by-products are utilised as a potential raw material in biotechnological processes for the production of energy and high-value products as they contain nutrients for the growth of microorganisms. These wastes can provide numerous high energy and useful products, such as biodiesel, bioethanol, multitudes of biochemicals, biogas and biofertilisers [7]. Biogas from these wastes is one of the most sought after and is a traditional technology. The abundance of biomass and sewage is the crucial factors driving the production of biogas [8]. Several countries have dedicated crops for biogas production. It is produced by anaerobic digestion of biomass. The controlled anaerobic process leads to primarily a gas mixture of 55–75% methane, 25–45% CO_2_. This mixture is called biogas. It can also contain a trace amount of H_2_S if the feed has sulphur in it. The biogas production process involves hydrolysis, acidogenesis, acetogenesis and methanogenesis [9]. The presence of methane, a high-energy molecule (calorific value of 55 MJ/kg), makes biogas a very attractive energy product.

**Fig. 1:**
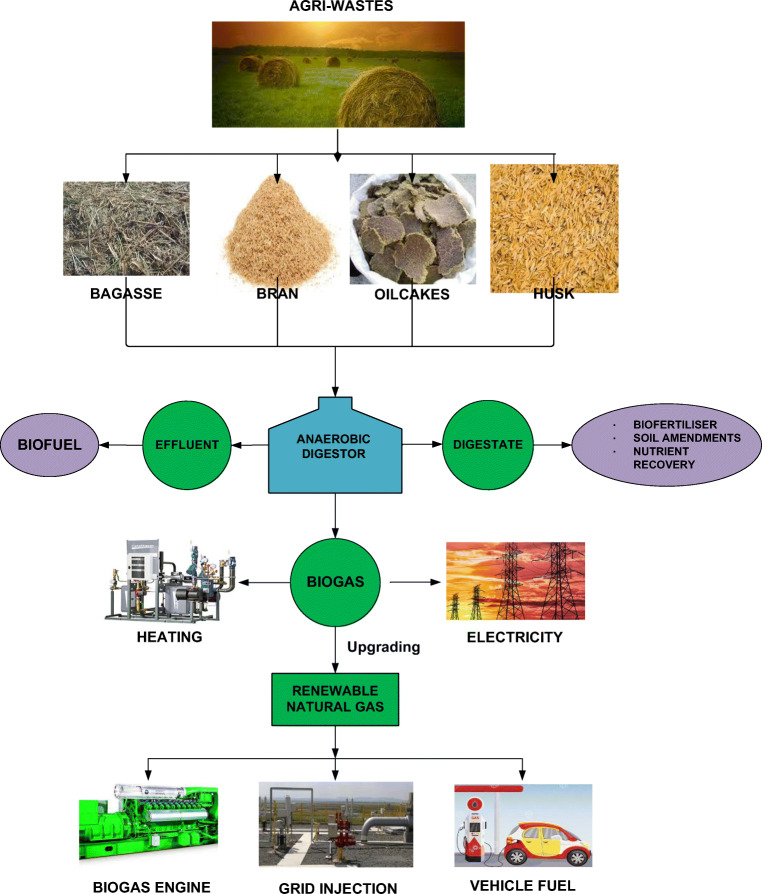
Agri-waste to biogas

Anaerobic digestion (AD) is also so advantageous that the digestate remained after the process also has multiple uses such as manure for the soil and feedstocks to aquaculture. AD offers several advantages compared to aerobic digestion (breakdown of organic matter in presence of oxygen, similar to activated sludge process, composting, etc.) including less energy requirements, less expensive, less sludge production and stable digestate along with production of energy [10, 11]. It, however, suffers from operational and start-up issues. Notwithstanding the disadvantages, AD has grown phenomenally in the last two decades. The biogas can be easily transformed into electricity, heat or fuel as per the requirements, and the technology has matured a lot [12]. In fact, several of the UN SDGs can be moderately or solely accomplished through biogas production. The biogas industry has the potential to not only manage all the wastes but also can mitigate food insecurities, fertilise the soil, improve air quality, protect water bodies and so on [13]. Some of the impacts could be direct, and some could be indirect. For example, replacing fossil fuels such as coal, petrol and diesel with biogas could greatly minimise GHG emissions. Biogas is the cornerstone of circular economy concept by improving the industries’ sustainability. Many of the industries such as paper mill, sugar mill and distilleries generate biogas and meet their own power requirements. Since urbanisation is a new normal, the waste generated from a highly urbanised population can be most effectively managed by biogas production [14, 15]. Figure 1 demonstrates in brief the potential of Agri-wastes to biogas production.

**Fig. 2:**
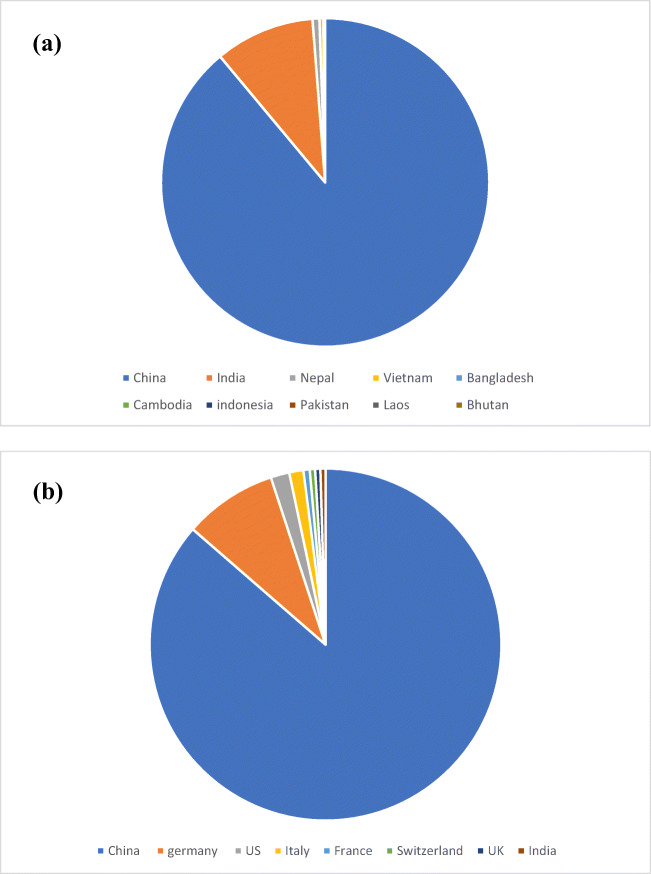
Distribution of small-scale digesters. **a** Asian regions, **b** across the globe [13]

**Fig. 3:**
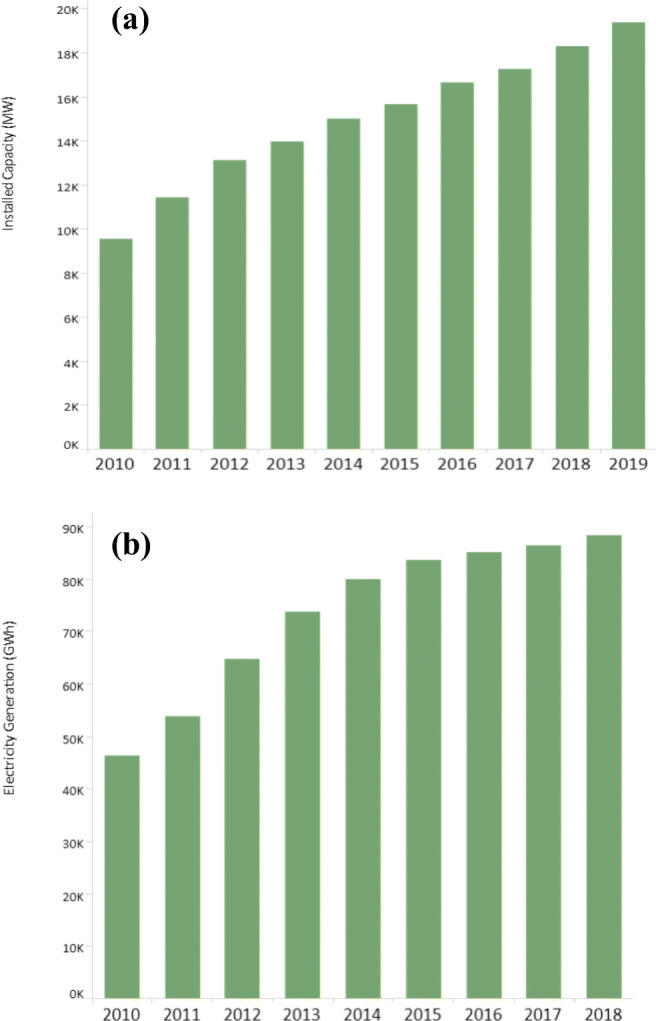
Growth of the biogas industry in the last decade in terms of **a** installed capacity and **b** electricity generation [16]

Small-scale biogas digesters are a very significant part of rural areas in developing countries. A rough estimate suggests that the total number of these digesters is around 50 million. Figure 2 shows the distribution of these digesters in various Asian countries. China leads the race with 84% of digester [16, 17]. Apart from small digesters, there are large-scale digesters producing electricity. They are usually in combined heat and power (CHP) mode. It is estimated that approximately 132,000 such digesters are being operational in the world. The percentage distribution of these digesters in the prominent region is shown in Fig. 2b. As can be seen, Germany is the leader in Europe with 10,000 and more digesters. Figure 3 shows the growth of the biogas industry in the last decade in terms of installed capacity and electricity generation. Another digester in vogue is for biogas upgrading. It will be seen in section 8.2, biogas upgrading though a recent phenomenon is rapidly becoming an integrated part of the AD systems. The most common upgrading is biogas to biomethane, but other valuable products can also be generated by biogas upgrading. As per the world biogas association (WBA) report, 700 plants upgrade biogas to biomethane globally [13].

Oil cakes are one of the common Agri-wastes, as it is a foremost product of the oil extraction from oilseeds [18]. Traditionally, the two methods used for oil extractions are mechanical extraction or solvent extraction. The former uses a mechanical device such as a screw press, and the later use solvents, hexane being the most common. The by-product obtained by pressing is termed as oil cake, and that by solvent extraction is labelled as oil meal [18]. The oil cake is incredibly rich in nutrients [19] and can be classified into two types, edible and non-edible. The cakes produced during the processing of edible oil-bearing seeds are edible oil cakes with a high protein content ranging from 15 to 50% [20]. The variability in composition mainly depends on plant growth conditions, seed quality and oil extraction methods [21]. The edible oil cakes are generally used as animal feeds based on their rich protein contents.

On the other hand, the oil cake resulting from the non-edible seeds that cannot be used as animal feeds owing to the existence of toxic compounds, and other impurities are distinguished as non-edible oil cakes [18]. Most of the non-edible oil cakes such as neem, castor, mahua and karanja cakes are used as organic fertilisers, due to their N, P and K contents. A massive amount of oil cake is produced every year according to Food and Agricultural Organisation (FAO)’s Food Outlook November 2020 report the global production of oil cakes and meal is forecasted to be 158.7 million tonnes globally in 2020–2021 which is similar to production in 2018–2019 which was 158.3 million tonnes [22]. Recently, biogas productions from these cakes are gaining traction, resulting in more and more information on this innovative research in biogas production. This work was conceived to collate all the information on biogas production from oil cakes and critically analyse the studies for the benefit of the academic and scientific community. It reviews biogas from both edible and non-edible oil cakes, discusses biogas upgrading and finally explores the economics and future perspectives of this route.

## Edible oil and the oil cakes

### Edible oil

Edible oils, extracted from the various plant, animal and synthetic sources, are composed of 96% triacylglycerides and different fatty acids. There is a wide variety of cooking oils from plant sources such as olive oil, palm oil, soybean oil, rapeseed oil, corn oil, peanut oil and other vegetable oils, extracted from the seed or fruit of the plant. Vegetable oil has one of the highest trade shares (40%) of the production of all agricultural commodities [23]. Major producers and exporters of oilseeds are the USA, Canada, Australia, Brazil and the EU. Though soybeans are the most produced type of oilseed, the world’s leading vegetable oil is palm oil. Indonesia and Malaysia are the two-leading exporter of palm oil in the world. Currently, Brazil and the USA are dominating the soybean seed production worldwide. In 2019–2020, soybean seed production worldwide was 337.9 million tons followed by rapeseed (69.2 million ton) and sunflower seed (56.7 million ton). The annual production of major global oil crops, as highlighted in the Food and Agricultural Organisation’s (FAO) biannual report on global food markets, is presented in Table 1.

**Table 1:** Global production of major oil crops

Oil seeds	2018–2019 million tons	2019–2020 million tons	Percentage change	Plant source	Oil cake source
Soybeans	365.6	337.9	− 7.6	*Glycine max*	Seed
Rapeseed/mustard	73.1	69.2	− 5.2	*Brassica napus*	Seed
Cottonseed	43.4	42.6	− 2.0	*Gossypium herbaceum*	Seed
Groundnuts	40.7	42.4	4.1	*Arachis hypogaea*	Seed
Sunflower seed	53.6	56.7	5.8	*Helianthus annuus*	Seed
Palm kernels	18.1	18.2	0.4	*Elaeis guineensis*	Kernel
Copra/coconut	6.0	5.5	8.2	*Cocos nucifera*	Endocarp

FAO Food Outlook, June 2020, http://www.fao.org/3/ca9509en/ca9509en.pdf

According to FAO, per capita, dietary consumption of vegetable oil is projected to grow at 0.9% per annum in the coming decade compared to 2% per annum growth observed between 2009 and 2018. China and Brazil will contribute significantly to this as the per capita oil food availability is going to be comparable to developed countries.

### Edible oil cakes

Globally, the crushing of oilseeds into oil cakes or oil meals and oil dominates total usage. The international edible oil cake market is primarily dominated by soybean cake, rapeseed cake, groundnut cake, sunflower cake, cottonseed cake, copra cake, etc., [23]. The demand for crush will increase faster than other uses, and by 2028, around 90% of soybean and 86% of world production of other oilseeds will be crushed [22]. As crushing operation depends on labour costs, transport costs, trade policies and infrastructure, the Chinese imported soybean crush is expected to increase about 31% of the world’s other soybean crush. However, the soybean crush of current decade expands only by 61 metric tons, which is well below the 111 metric tons expansion of the previous decade. According to FAO, the global edible oil cakes or oil meal production in 2019–2020 is expected to decline significantly to 348.9 million tons, corresponding to around a 6% reduction from the previous season’s record level. The decline is attributed predominantly to the drops in soybean and rapeseed production due to adverse weather condition, harvest area contractions, reduced yield and poor harvest.

On the other hand, consumption of these cakes is seen to keep increasing, though at a below-average rate, due to COVID-19 mediated temporary lockdowns imposed in numerous countries. Therefore, global oil cakes or oil meal supplies are estimated to decrease by 3.7%. However, global stocks of oil cakes or oil meals are expected to fall to multi-year lows, resulting in a substantial drop in stocks-to-use ratios [22].

The chemical composition and nutrient availability of the oil cakes determine their utility. Therefore, the chemical compositions of oil cakes have been extensively investigated by many researchers. Depending on the pre-processing like removal of hulls, the fibrous outer covering enclosing the seed (dehulling) and mode of oil extraction, the chemical composition of oil cakes varies. For example, the mechanically pressed oil cakes contain more residual oil than the oil cakes produced by solvent extraction method [23]. The chemical compositions of some of the major global oil cakes mostly generated during solvent extraction method are shown in the Table 2.

**Table 2:** Composition of major oil cakes

Oil cakes	Dry matter (%)	Protein (%)	Fibre (%)	Carbohydrate (%)	Fat (%)	Ash (%)	Calcium (%)	Phosphorous (%)
Soybean cakes	84.8–90.3	47.5–51.8	5.1–17.8	23 ± 0.6	0.8 ± 0.1	6.4–7.3	0.1 ± 0.03	0.65 ± 0.04
Rapeseed cakes	89.8–90.7	38.5–42.8	3.5–12.1	32 ± 0.2	4 ± 0.1	7–9.9	0.04 ± 0.01	1.1 ± 0.01
Cottonseed cakes	91.5–94.3	40.3–41.5	14.7–15.7	26.5 ± 0.5	5 ± 0.8	6.5–6.8	0.3 ± 0.01	0.1 ± 0.01
Groundnut cakes	90–92.6	45.6–49.5	5.3–8.3	14 ± 0.1	2 ± 0.5	4.5–5	0.1 ± 0.01	0.7 ± 0.04
Sunflower seed cakes	91–93	34.1–35.6	13.2–28.4	22.5 ± 0.5	1.5 ± 0.3	6.6–7.4	0.25 ± 0.05	1.2 ± 0.1
Palm kernel cakes	90.8–93	17.5–18.6	11.9–37	45 ± 0.5	7 ± 0.4	4.5–4.8	0.3 ± 0.01	0.8 ± 0.05
Copra cakes	88.8–89.9	20.9–25.2	10.8–11.5	42 ± 0.4	7.5 ± 0.5	5.5–6	0.05 ± 0.03	0.6 ± 0.07

Summarised from Ramachandran et al. (2007), Sivaramakrishnan and Gangadharan (2009)

Soybean cake has rich protein content than most other oil cakes and is an excellent source of amino acids, as shown in Table 3 that presents amino acid compositions of some important oilseed cakes. Approximately 15% of carbohydrates in soybeans are oligosaccharides like sucrose and raffinose. In addition, small quantities of anti-nutrients such as lectins, saponins and phytates were reported in soybean cakes [23]. The rapeseed oil cake has comparable amino acid balance as that of soybean cake. Around 14.5% of carbohydrates in rapeseed cake are pectins. The anti-nutrients reported in rapeseed cakes are tannins, erucic acid, sinapine and phytates. Cottonseed cake has a protein content of about 40–41%, the fibre content of 14–15%, and comparatively low methionine, tryptophan and histidine levels. The presence of toxic metabolite, gossypol in cottonseed cake, makes it difficult to use it as feed ingredients. Groundnut oil cake has a high protein content of 45–49%; the low fibre content of 5–8% is rich in arginine (5–11%), but low in methionine and tryptophan. These cakes are prone to contamination by aflatoxins, a fungal toxin from *Aspergillus flavus*, limiting the utilisation scope of the cakes [23]. Sunflower oil cake has about 34–35% crude protein and fibre of 13–28% is high in arginine, leucine but low in alanine and tryptophan. No anti-nutritional factors are reported in sunflower, but the presence of a polyphenolic compound, chlorogenic acid, inhibits hydrolytic enzymes activity. Palm kernel cakes with 17–18% of crude proteins have the lowest protein content and the highest fibre content among all the other oil cakes and contain high levels of galactomannans [20]. It is deficient in alanine and serine. Due to the high fibre content, the digestibility of the cake is low for monogastric animals [24]. Copra cake has a protein content of about 20–25% and highest fat content (8%) as it contains high levels of residual oil composed of short-chain saturated fatty acids. It is tryptophan deficient, low in alanine and serine but high in arginine and valine.

**Table 3:** Amino acid **c**omposition of major oil cakes

Amino acids (% of crude protein)													
Oil cakes	Ala	Arg	Gly	His	Ile	Leu	Lys	Met	Phe	Ser	Thr	Trp	Val
Soybean cakes	2 ± 0.15	3.6–7.4	2–4.5	1.3–2.4	2.1–4.6	3.8–7.8	3–6.1	0.7–1.4	2.4–5.5	2.2 ± 0.4	2–3.8	0.8–1.3	2.1–5.2
Rapeseed cakes	1.5 ± 0.2	2.5–6.4	2.6–4.9	1.2–2.6	1.6–3.8	2.8–6.3	2.4–5.4	0.7–1.7	1.6–3.8	1.5 ± 0.5	1.9–4	–	2–4.7
Cottonseed cakes	4 ± 0.5	11.1 ± 0.4	4.5 ± 0.4	2.2 ± 0.2	3.2 ± 0.2	5.9–6.6	4.1 ± 0.3	1.3 ± 0.5	5.4 ± 0.3	4.5 ± 0.2	3.2 ± 0.4	1 ± 0.1	4.5–5.1
Groundnut cakes	1.5 ± 0.3	5–11	5.5 ± 0.5	1.1–2.5	1.5–3	2.9–6.1	1.5–3.6	0.4 ± 0.1	2.3–4.9	1.5 ± 0.2	1.1–2.8	0.4 ± 0.1	1.9–3.7
Sunflower seed cakes	1 ± 0.4	2.4–9.1	1.9–5.6	0.7–2.8	1.2–4.2	2–6.9	1–3.5	0.7–2.2	1.4–5.1	1.2 ± 0.1	1.2–3.4	1.1 ± 0.3	1.5–5.8
Palm kernel cakes	–	2.2–13.9	0.8–4.8	0.3–2.5	0.62–3.8	1.1–6.4	0.6–3.7	0.3–2.7	0.7–3.6	0.5 ± 0.1	0.2–3.5	0.2–2.8	0.9–5.7
Copra cakes	0.8 ± 0.01	2–11	0.9–4.2	0.4–2.1	0.6–3	1.2–6	0.4–2.5	0.3–1	0.8–4.1	0.9 ± 0.1	0.7–3	–	0.9–5.8

Summarised from Ramachandran et al. (2007), Sivaramakrishnan and Gangadharan (2009)

## Non-edible oil and oil cake

### Non-edible oils

Jatropha (*Jatropha curcas*), karanja (*Pongamia pinnata*), mahua (*Madhuca indica*), silk cotton tree (*Ceiba pentandra*), castor (*Ricinus communis*), etc. are the non-edible plants capable of producing oils. They are plentily available in several parts of the world and are inexpensive in contrast to the edible oils [25]. The mahua tree grows on a varied variety of soils but flourishes the greatest on sandy soil. The species is drought-resistant, strong light demander and readily suppressed under shade. It is mostly grown in central India and is one of the most important trees for the tribal. The castor bean plant, *Ricinus communis*, is a native to Ethiopia. It has now become widespread in the tropical and warm temperate regions, as well [26]. The crops can be easily grown in waste and futile land, making the land reclamation amenable. Table 4 details the oil contents of these plants.

**Table 4:** Oil contents of major non-edible plants

Sl. no	Oilseed generic name	Botanical name	Oil content (%)	References
1	Jatropha	*Jatropha curcas*	30–40	[27]
2	Mahua	*Madhuca indica*	35	[27]
3	Karanja	*Pongamia pinnata*	27–39	[28]
4	Castor	*Ricinus communis*	48–60	[29]
5	Neem	*Azadirachta indica*	20	[30]
6	Polanga	*Calophyllum inophyllum*	65	[31]

**Fig. 4:**
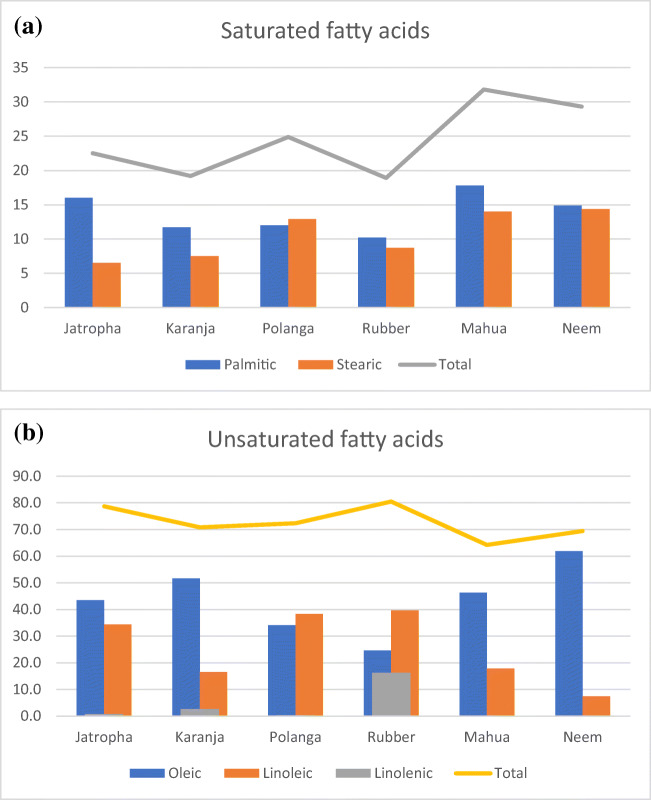
Fatty acid composition of major non-edible oils. **a** Saturated fatty acids and **b** unsaturated fatty acids

The saturated and unsaturated fatty acid composition of several non-edible oils is presented in Fig. 4a and b [32–36].

The oilseeds are mostly grown in southern Asia, Africa, Brazil and China. Some of them are also distributed in the tropical and temperate climate. The typical region can be taken as India, Thailand, Malaysia, north-eastern Australia, etc. Almost all the oils have demonstrated capability for biodiesel production and biorefinery. There are various advantages of biodiesel from non-edible oils, such as the ready availability of the oils, good combustion efficiency, liquid nature portability and renewability [37]. Consequently, over the years, there has been a gradual shift from edible oils to the low-cost non-edible feedstock.

### Non-edible oil cake

In a similar manner as edible oils, the oil extraction from the non-edible oil seeds leaves behind the cake. The plant being non-edible renders cake also not directly utilisable as organic manure or a cattle feed. The cakes include contaminated materials like chromenoflavones (karanja), phorbol esters (Jatropha), strong odour (neem) and ricin (castor). The disposal of these cakes is a challenge being faced by the agriculture industry [38]. The cakes being organic are susceptible to microbial action in the open atmosphere. Microbial activities lead to the generation of several gases, such as CH_4_, N_2_O, H_2_S, NH_3_, CO_2_ and volatile organic compounds (VOCs). There are other health and environmental hazards [39].

Various researchers have tried eco-friendly ways to dispose of these wastes. Das et al. (2014) studied jatropha cake as biosorbents in treating wastewaters containing reactive red dye [40]. They reported the highest dye adsorption capacity under the neutral condition and room temperature. The optimised adsorption period was 6 h. They also tried various isotherms and deduced that Redlich–Peterson and Sip isotherms represented the adsorption better than other isotherms. Equally, Bose et al. (2011) successfully applied the jatropha cake as an adsorbent for Cr(IV) removal from the wastewaters [41]. Another work by Upendar et al. (2013) reported karanja cake as adsorbents for CO_2_ capture [42]. Irfanudeen et al. (2015) reported the use of cake as a biogenic substrate [43]. Francis et al. (2005) studied the application of the jatropha cake as manure. They concluded that it has more nutrients compared to the chicken and cattle manure [44]. Another study found that the jatropha cake is beneficial as a good soil amendment or a fertiliser [45]. Similarly, mahua seed cake was also applied as manure alone as well as in combination with other cakes and ammonium sulfate [46]. However, Hirota et al. (1988) cautioned that the cake application as fertiliser could lead to biosafety issues [47]. They theorised that the phorbol esters present in the Jatropha cake could boost skin tumour. The jatropha cake was also tested for its application in briquette production [48]. They reported that the jatropha cake amended briquets were combusted fully in 35 min at high temperatures. Mahanta et al. (2008) applied the jatropha cake as a substrate to manufacture industrial enzymes, like protease and lipase. The microbes used was *Pseudomonas aeruginosa* [49]. The mahua cake was also reported to be used in the preparation of various domestic items such as detergents and shampoo [50]. There are other studies on the application of these cakes, such as fermentation of the seed cake to produce ethanol [51]. However, anaerobic digestion of these cakes could be the most feasible solution resulting in energy and taking care of their disposal issues.

## Applications of oil cakes

**Fig. 5:**
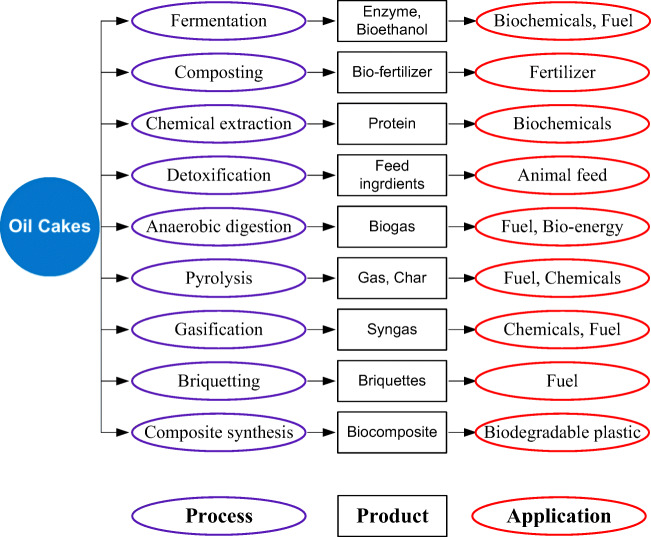
Various applications of oil cakes. (conceptualised from Jingura and Kamusoko (2018))

Oil cakes have been widely used to produce chemicals, fuels, industrial enzymes, fertilisers and biochemicals like vitamins and antibiotics [20]. After detoxification or in case of non-toxic varieties, they have been used as a feed supplement. The processes to transform the oil cakes into valuable end-products are very diverse in nature, including physical, chemical, thermochemical and biological methods [52]. Some important applications of oil cakes are illustrated in Fig. 5. Oil cakes are used as substrates mainly as carbon and nitrogen source to produce varieties of biochemicals including enzymes, antibiotics, vitamins and bio-pesticides either through solid-state fermentation (SSF) or submerged fermentation (SmF). For example, when rice straw substrate is supplemented with soybean cakes, rapeseed cakes, sunflower seed cakes, cottonseed cakes, etc., the mushroom yield increases about 50–100% compared to the un-supplemented substrate [20]. There is an increasing global trend in using edible oil cakes as livestock feed mainly for poultry, non-ruminants and aquaculture. However, several physicals, chemical or biological pre-treatment steps are essential to remove anti-nutrients or toxic components and to enhance the nutritional value of oil cakes [23]. The organic fertiliser potential of oil cakes has been demonstrated widely after subjecting them to composting for biological decomposition. Bioethanol, an alternative to fossil fuel, is produced by the fermentation of the carbohydrate-rich oil cakes. However, for enhanced bioethanol production, enzymatic hydrolysis pre-treatment step is needed primarily to hydrolyse the recalcitrant components (cellulose, hemicellulose) of the oil cakes [53]. When oil cakes are subjected to pyrolysis, the cellulose, hemicellulose and lignin contents undergo thermal degradation in the absence of oxygen to generate energy rich products like bio-oil, char and gases [54]. Similarly, the gasification process converts the carbon rich oil cakes into a combustible gas known as syngas. The fibre contents of the oil cakes make them suitable raw materials for further reprocessing by mixing them with plastics to produce bio-based composite materials that are eco-friendly, cheap, low density and biodegradable [55]. Furthermore, the densification of oil cakes by briquetting techniques results in bio-briquettes, the compact concrete composites that are easy to collect, store, transport and use as an energy source via combustion. Among all the applications of oil cakes, biogas production is one of the most promising applications and therefore, has been discussed in detail in the following sections.

## Biogas production from edible oil cakes

### Biogas production from sunflower oil cake

Sunflower oil globally is seeing an upward trend in recent times with Ukraine and Russia being the primary producers. Major consumers of sunflower oil are food, cosmetic and pharma industries. Sunflower oil extraction leaves behind a highly nutritious deoiled cake which is primarily used as animal feed and fertiliser [19, 56]. Furthermore, this inexpensive feedstock can act as an excellent substrate for the production of biogas. Various research papers have focused on the effects of pre-treatment methods, inoculum type and operation parameters on the production of biogas from sunflower oil cake. Anaerobic digestion is the preferred method for utilisation of deoiled cakes to produce biogas [57]. However, the lignin content in the biomass influences the efficiency of bioconversion.

Various physicochemical pre-treatment methods have been used to overcome the low accessibility of biodegradable organic fraction in lignocellulosic biomass. Biogas production potential of sunflower oil cakes has been reported to be in the range of 186 to 215 mL CH_4_/g volatile solids which is relatively low and corresponds to only 40% of the organic solid [58]. This low conversion efficiency could be attributed to lesser availability of hollocelluloses interlinked as a complex polymer of cellulose, hemicellulose and lignin to microorganisms. Efficient pre-treatment methods help in breaking down the linkages between these complex polysaccharides within the lignocellulosic network to make the fermentable sugars available to the microorganisms.

Effect of combined treatment of high temperature and dilute acid on the methane production efficiency from sunflower oil cake has been reported. Monlau et al. (2013) have tried different sulphuric acid concentrations and temperature range combination on the solubilisation of organic carbon, sugars and proteins [58]. They found that pre-treatment combination of 1% H_2_SO_4_ and 170 °C temperature gave the best result and increased the methane yield to 302 ± 10 mL CH_4_/g volatile solid (VS) compared to the untreated sample which produced 195 mL CH_4_/ g VS. Increasing the temperature further did not increase the yield due to Maillard reaction and lowering of soluble carbohydrate concentration [58].

Another study evaluated the methane yield of sunflower oil cake using thermochemical pre-treatment method. Lime, sodium hydroxide, sulphuric acid and sodium bicarbonate were the four chemical compounds used for the pre-treatment at a concentration of 25% (*w*/w) of the substrate dry weight and 20 g/L substrate concentration followed by thermal treatment at 75 °C. Results showed that the solid fraction generated with lime treatment only showed a 25% higher methane yield of 130 mL CH_4_/g chemical oxygen demand (COD)_added_ than untreated solid material. However, the overall methane yield from solid and liquid fraction showed no increase from untreated sunflower oil cake [59].

Hydrothermal pre-treatment has been shown to increase the accessibility of cellulose by reducing the crystallinity and increasing the surface area of lignocellulosic biomass apart from being environmentally friendly with less corrosion and no catalyst requirement [60, 61]. It has been used to increase methane production from sunflower oil cake where comparisons were made after treatment with four different temperatures of 25, 100, 150 and 200 °C. It was observed that under batch fermentation in mesophilic conditions, pre-treatment of 100 °C resulted in highest methane yield of 105 ± 7 mL CH_4_/g COD_added_ for solid fraction and 310 ± 4 mL CH_4_/g COD_added_ for the liquid fraction. However, the authors noted that yield was only 6.5% higher compared to pre-treatment at 25 °C [62].

Fernández-Cegrí et al. (2012) have shown that ultrasonication pre-treatment can improve anaerobic digestion under mesophilic condition [63]. Two percent (*w*/*v*) substrate was treated with five ultrasonic pre-treatment conditions in a range of 24,000 kJ/kg TS and 16.6 min to 597,600 kJ/kg TS and 331.2 min, while maintaining a constant sonication frequency (20 kHz) and ultrasonic power (120 W). The 24,000 kJ/kg TS gave the best result among all the treatments with a methane yield of 220 ± 11 mL CH_4_ STP/g COD_added_, which was also 53.8% higher than untreated oil cakes [63]. In another study by this group, they tried to understand the role of ultrasonication pre-treatment of sunflower oil cake semi-continuous mode anaerobic digestion. The laboratory scale stirred tank reactors were operated in mesophilic temperature of 35 °C and ultrasonication was done at 24,000 kJ/kg TS with constant sonication frequency of 20 kHz and ultrasonic power of 120 W. Effect of different inoculum on methane production was also tested by using floccular inoculum from anaerobic reactor treating waste activated sludge and another granular inoculum from industrial up-flow anaerobic sludge blanket (UASB) reactor treating brewery wastewater. The overall methane production was 13% higher in the case of granular inoculum from UASB operating with brewery wastewater. In addition, pretreated samples showed 1.9% higher methane production [63].

This study points at another very critical aspect of anaerobic digestion of sunflower oil cake for biogas production i.e. microbial inoculum. Anaerobic digestion for production of biogas relies on the activity of the groups of microorganisms which carries out hydrolysis followed by acidogenesis and methanogenesis. The optimum diversity and abundance of each group of microorganisms carrying out different steps determine the overall stability and efficiency of the process. The study by Rincón et al. (2011) showed the variability in methane production from sunflower oil cake when three different anaerobic consortia were used [64]. The three inoculum sources were (i) granular inoculum from an industrial reactor treating soft-drink wastewater, (ii) flocculent biomass from a full-scale reactor treating biosolids generated in an urban wastewater treatment plant and (iii) granular biomass from an industrial reactor treating brewery wastes. The biochemical methane production (BMP) test showed that granular biomass from an industrial reactor treating brewery wastes showed 7.5% higher compared to inoculum (ii) and 31.1% higher from inoculum (i). Polymerase chain reaction (PCR) amplification of 16 s rRNA gene followed by molecular fingerprinting was used to identify the bacterial and archaeal communities of all the three inoculums. The results suggested that archaeal communities corresponded to methane producing archaea of Methanosarcinales and Methanomicrobiales orders [64].

Apart from the inoculum type, it has also been shown that inoculum-substrate ratio also plays a role in anaerobic digestion sunflower oil cake in batch mode under mesophilic condition. A range of inoculum–substrate ratios (ISRs) of 3.0, 2.0, 1.5, 1.0, 0.8 and 0.5 expressed as volatile solids affected the methane yield and biodegradability. The study showed that ultimate methane yield decreased considerably from 227 ± 23 to 107 ± 11 mL CH_4_/g VS_added_ when the ISR decreased from 3.0 to 0.5. It establishes the influence of the ISR on the methane yield coefficient. The biodegradability of the waste also decreased from 86 to 41% when the ISR varied from 3.0 to 0.5, but a closer observation of net total ammonia nitrogen yield showed that lowering the ISR is not influencing the hydrolytic-acidogenic stage but the methanogenic process in lower ISR range of 0.5 and 0.8 [65]. Another research with the same range of ISR showed was used and two kinetic models for substrate (volatile solids) degradation and methane production were obtained. The kinetic constants for volatile solids degradation (K1) and methane production (K2) decreased from 0.54 ± 0.09 to 0.32 ± 0.03 day^−1^ and from 0.36 ± 0.04 to 0.16 ± 0.03 day^−1^, respectively, with the decrease in ISR from 3.0 to 0.5, showing the occurrence of an inhibition phenomenon by substrate concentration [66].

The larger surface area of lignocellulosic biomass has been shown to increase the initial degradation rate [67]. Reducing the size of feedstock can increase the surface area and aid in microbial degradation, and clogging of the digester can be avoided. However, the lignocellulosic composition of different size fractions can be different, which may affect the anaerobic digestion. Rubia et al. (2011) analysed the effect of particle size and chemical composition on biogas production from sunflower oil cake by using different particle sizes of 0.355–0.55 mm, 0.710–1.0 mm and 1.4–2.0 mm in diameter. Interestingly, the larger particle size range produced the highest methane yield 213 ± 8 mL CH_4_ g^−1^ VS_added_. Authors suggest that varied lignocellulosic composition in different fractions and evolution of volatile fatty acid (VFA) like propionic acid in the smallest particle size could be the reason for lower biogas production [68].

The various operational parameters of anaerobic digester like hydraulic retention time (HRT) and organic loading rate (OLR) also have been shown to affect the hydrolytic-acidogenic phase of anaerobic digestion of sunflower oil cake. Six OLR (ranging from 4 to 9 g VS L^−1^ day^−1^) for four HRTs (8, 10, 12 and 15 days) were tested in two stage anaerobic digestor operating in mesophilic range. Results suggest that all range of HRTs and OLRs did not have a significant variation on the hydrolysis step, which was between 20.5 and 30.1%. However, different OLRs influenced the degree of acidification with OLR of 6 g VS/ (L day) showing the highest value of 83.8% [69].

### Biogas production from other edible oil cake

Soybean cake, coconut oil cake, mustard oil cake, palm kernel cake, groundnut oil cake, cottonseed cake, canola oil cake, olive oil cake and rapeseed cake are the other most prominent edible oil cakes that are produced in significant quantity across various geographical regions. Though large quantities of these oil cakes are generated annually, most of the edible oil cakes are used as animal feed and substrate for enzyme production due to their high nutritional value [18]. It has also been highlighted in section 5, and hence, limited studies are available on the biogas production from these oil cakes. Most of these oil cakes have been used with other substrates like cattle manure for co-digestion.

#### Olive cake

Olive cakes consisting of seed particles and the fleshy parts are the by-product of olive oil extraction. Mediterranean region is the largest producer of olive oil. Because of the health benefits of olive oil, the consumption is steadily increasing over the years. Only 21% of the olive is the oil itself, resulting in the production of a lot of waste during the oil processing. A commercial biogas industry in Norway has checked the biogas production potential of olive cake as a substrate for its existing biogas plant. The data shows that with substrate inoculum ratio of 0.32, the cumulative yield of biogas from olive cake was 705 ml. It increased to 1226 mL when the substrate inoculum ratio was 0.64. The retention time used was 63 days [70].

Co-digestion of olive oil cake with animal manure like pigeon waste and rabbit waste has been reported. Anaerobic digestion was performed at 30 ^o^C for 40 days with varied ratios of animal manure, and olive oil cake and result indicates that combining olive waste with animal manure could produce biogas in a sustainable manner [71].

#### Palm oil cake

Solid-state fermentation of palm decanter cake was evaluated using different ratio of biomass and inoculum. The cumulative methane production of 130 mL CH_4_/g VS was observed for decanter oil at biomass inoculum ratio of 2:1. Total methane production using solid-state anaerobic digestion was 41 m^3^ CH_4_/t which was lower than the other solid waste generated from palm tree like fruit branches, palm press fibre [72]. Another study also used palm decanter cake for biogas production in 0.5-L batch reactor operating at 37 °C and pH 7. Production of methane and hydrogen gas was studied using varying organic loading 2.5–10% *w*/*v*. Two different inoculum i.e. sludge combined with the indigenous microbe and only indigenous microbes were used, and their performances were compared in terms of methane production. The result suggested that source of inoculum strongly affects the gas composition after anaerobic digestion, CH_4_ was the predominant biogas composition and no H_2_ was observed in the combined seed fermentation. In contrast, it was present in indigenous microbe fermentation. Sludge inoculum showed better biogas yield because of the presence of methanogenic bacteria [73].

#### Mustard oil cake

Co-digestion of mustard oil cake with cattle dung has been reported to produce biogas. An increase in biogas production of 13.38%, 25.27%, 39.16%, 52.26% and 63.44% was observed when 10%, 15%, 20%, 25% and 30% of mustard meal/cake was added to cattle dung as the substrate for anaerobic digestion, respectively. In the case of 30% mustard cake addition, an increase in volatile solids destruction of 12.2–13.08% was achieved, with corresponding gas production of 4591 ml day^−1^ compared to 2809 ml day^−1^ biogas production with only cattle dung. However, based on the mustard cake availability, an optimum addition of 20% was recommended. The manurial value of the digested sludge was found to be very good, and with capillary suction time (CST) of 320–394 s, good waterability of the digested sludge was confirmed [74].

#### Cotton oil cake

Biogas production has been done from cotton waste, including cotton oil cake. The biochemical methane potential test results point out that 78 ml of methane can be produced from 1 g of cotton oil cake in basal medium after 23 days of anaerobic digestion [75].

#### Rapeseed oil cake

Co-digestion of rapeseed oil cakes in the range of 1–5% with waste activated sludge (95–99%) for biogas production was studied. Microwave-assisted pre-treatment of substrates and HRT of 20-22 days produced a double amount of biogas with 10–14% more methane than only activated sludge biomass [76]. Another study investigates the effect of solid concentration on biogas production from rapeseed oil cake. The solid concentration of 10%, 15%, 20% and 25% of total solids were used for laboratory scale 2 L batch reactor with a retention time of 30 days. Best results were obtained with 20% solid concentration, which gave the highest yield of biogas under the experimental conditions [77].

## Biogas productions from non-edible oil cakes

### Biogas production from Jatropha oil cake

Literature suggests that *Jatropha curcas* is deemed to be the most suitable contenders for biofuel and bioenergy [78, 79]. Ram Chandra et al. (2006) studied biogas generation from jatropha [80]. Anaerobic digestions of the seed cakes were conducted at 37 °C in the laboratory conditions. A 5 L glass fermentor was used. They combined the cakes with various combinations of cattle dung. The gas production was estimated at different hydraulic retention times (HRTs). As high as 265 L/kg weight of cakes were obtained with the methane concentration around 65%. They estimated that this would result in 2550 million cubic metres of biogas. In another study on anaerobic digestion of jatropha, Ram Chandra et al. (2012) found that biogas production rate stabilised within a short time [81]. The substrate temperature changes mildly during the digestion process. Methane content was on the higher side with 68% methane found at HRT of 20 days. The cumulative biogas yield is around 180 m^3^. They observed higher values of methane fraction from oil seed cake in comparison with the biogas from cattle dung. They also found the specific methane production yield from total solid and volatile solid for both the cakes. The values obtained were 0.097–0.47 m^3^/kg TS and 0.104–0.506 m^3^/kg VS. Shilpkar et al. (2009) tried biomethanation of jatropha along with the buffalo dung. The experiments were conducted in a 5-L digestor for 6 months [82]. Almost 140% more biogas production was recorded for the oil cake and dung combination than the dung alone. Methane content was also observed to be very high (71%). Other positive results from the digestion were high nutritive value of the test slurry and 93% reduction in COD. Statistical analysis by authors unequivocally proves the significantly high nutrition obtained when the seed cake and dung were combined.

Schmidt (2011) investigated the anaerobic digestion of jatropha cake in the presence of iron additive (IA) on gas quality [83]. They also studied the process stability during the OLR increase. They found out that the jatropha cake has the potential to be consumed as a singular substrate for biogas generation up to an OLR of 2.4 g VS/(L.day). The IA enhanced the biogas quality by reducing the H_2_S content in the biogas. The H_2_S concentration in biogas was obtained as low as 258 ppm. The results indicate the high buffering capacity of jatropha cake facilitating anaerobic digestion easily. It was estimated that 3 kg of jatropha cake might yield 683 L of methane equivalent to 22 MJ of energy. The calorific value of jatropha oil is 40.7 MJ/kg, and it produces 3 kg of cake. The authors estimated anaerobic digestion of the cake could increase the energy efficiency of the process by 50%.

Visser and Adriaans (2007) obtained 0.5–0.6 m^3^/kg methane production using jatropha cake. The cakes were obtained using the cold-pressing of jatropha seeds [84]. They used four varieties of cake depending on nozzle size and the hull content. As high as 0.95 m^3^ of biogas/kg of dry matter was noted, with a high methane content of 83%. Their studies hypothesised that a large-scale biogas plant would be able to generate biogas with an LHV of 18–22 MJ/kg. They further found that the H_2_S concentration in all samples was less than 0.18 mg/m^3^. It was lower than the detection limit of chromatograph used. Staubmann et al. (1997) reported 0.446 m^3^ of biogas/kg of dry cake, comprising 70% CH_4_ [85]. They used pig manure as inoculum. Raheman and Mondal (2012) stated jatropha cake generates more biogas in comparison the cattle dung [86]. Biogas production was 0.17 m^3^ at 20% TS of jatropha cake compared to 0.166 m^3^ in the case of cow dung slurry only. They tested at various total solids concentration and C/N ratio. They further noted that the nitrogen content in the anaerobically digested jatropha slurry improved by 5.9% compared to the cake alone. The biodigested slurry was used as a fertiliser, and it delivered superior growth of maise and tomato. They overwhelmingly concluded that jatropha cake is a good feedstock for biomethanation and the attendant benefits. It is one of the sustainable ways to deal with the disposal problems of jatropha cake. Singh et al. (2008) noted that biogas production from jatropha cake was much greater than that produced from the cattle dung [48]. It also had higher calorific value than cattle dung, owing to more methane in the biogas. Grimsby et al. (2013) experimented with jatropha cake, digesting it in an anaerobic batch reactor with 1% VS and 71-day incubation [87]. The digestion generated 289 L·of methane/kg VS. Methane concentration in the biogas was around 60 to 65% with energy yield as 4.7 MJ/kg VS. It was approximately 20% of the energy in the undigested jatropha press cake. They also opined that water needs and the slurry’s liquid form make anaerobic digestion of jatropha cake a viable technology, especially in rainfed areas. Sinbuathong et al. (2012) studied jatropha cake degradation using a two-stage anaerobic reactor conducting acidogenesis and methanogenesis separately [88]. They realised that the maximum methane yield was observed at an organic loading rate of 3.3 kg COD/m^3^ day. It corresponded to hydraulic retention times of 30 days for each stage. Their study revealed that high methane yield could be obtained from Jatropha cake in a two-stage anaerobic process without chemical addition for pH adjustment. The optimal dilution was found to be 1:20. They also recorded high COD degradation efficiencies. In a co-digestion study, Sen et al. (2013) experimented with jatropha cake and bagasse in the presence of Fe^2+^ [89]. They found that an optimum jatropha cake concentration yielded 66.4 mL*/*d biogas production rate (BPR) and 0.064m^3^*/*kg VS biogas. The co-digestion with bagasse improved the carbon/nitrogen of feed to 26.5 from 14 (jatropha cake alone), consequently yielding 0.136 m^3^*/*kg VS, more than 100% increase compared to jatropha cake alone. Further addition of Fe^2+^ to jatropha cake and bagasse mixture increased the biogas yield to 0.203 m^3^*/*kg VS, with a methane content of 66%. The study was conducted with 15 days of digestion time. The study is remarkable, showing the high potential of biogas generation from co-digestion of jatropha cake with other Agri-wastes.

Several researchers are also trying continuous anaerobic digestion of oil cakes. Singhal et al. (2018) used a pilot-scale continuous stirred tank reactor to digest jatropha cake with a total capacity of 40 m^3^ [90]. The cake was digested with cow dung (3:1) in the reactor to constantly produce biogas for 120 days. Within 5 days, the reactor started producing 20 m^3^ biogas per day. Jablonski et al. (2017) studied thermal and acidic pre-treatment of jatropha cake to enhance the efficiency of anaerobic digestion [91]. They hypothesised that pre-treatments could deactivate protease inhibitors and partially hydrolyse phytate. They found that although the pre-treatment altered the kinetics of anaerobic digestion, reducing protease inhibitor activity and phytate concentration, it did not increase the biogas production efficiency. It could be due to the fact that the pre-treatments did not target lignin and cellulose, which might make the anaerobic process inefficient.

### Biogas production from karanja oil cake

In a work on karanja cake, Barik and Murugan (2015) studied the anaerobic degradation of karanja cake in combination with the cow dung [92]. They worked on four different karanja and cow dung proportions, like 75:25, 50:50, 25:75 and 0:100 on a mass basis. They evaluated pH, temperature, hydraulic retention time (HRT) and carbon/nitrogen ratio (C/N). It was observed that the proportion of 25:75 produced the best results. Methane content was 73% and the slurry has a higher fertiliser value and was more non-toxic. Correspondingly, the biogas from the sample had a heating value of 27.5 MJ/kg, with an energy content of 6–6.5 kW/m^3^. Barik and Murugan (2015) further studied modeling of the process for prediction and optimisation of biogas production using artificial neural network (ANN) and the genetic algorithm (GA) [93]. The GA optimised results based upon ANN developed model for pH, digestion time and the C/N ratio of the samples were correlated with the experimental results. Similar results were observed by Kumar et al. (2013). They studied various combinations of karanja cake and cattle dung [4]. They found that the biogas obtained from pure karanja cake was comparable to those obtained from cow dung.

### Biogas production from other non-edible cakes

Apart from jatropha and karanja, various other non-edible oil cakes have been tested for biogas production. Lingaiah and Rajasekaran, (1986) studied biogas production from castor cake as early as 1986 [94]. They observed that with appropriate C/N ratio variations, a variety of wastes along with castor cake could gainfully be utilised for maximum gas output. Eighteen·3 L of gas output were obtained for a period of 6 weeks. Similarly, the effect of particle size, temperature, loading rate and stirring on biogas production from castor cake was studied in 5-L capacity single-stage fermentors at 30 and 37 °C [95]. They used four particle sizes in the range 0·5 to 2·0 mm, the volumetric loading rates from 4 to 12 g TS/L.day. They maintained the HRT constant as 15 days in all cases. They reported that both the rate and biogas production were higher with particles 2·0 to 1·4 mm and 0·5 mm, and less with particles of intermediate size. Besides, high temperature favoured the higher yield. The optimal loading rate realised was the lowest one, 4 g TS/ (L day). Bateni et al. (2017) studied the anaerobic degradation of alkali pre-treated castor cake [96]. It was found that pre-treatment did not enhance biogas production and the highest methane production was 252.1 L/kg VS obtained from the untreated seed cake. It could be that they have used the extreme temperature. Some optimal temperature would have resulted in higher methane production. The untreated cake had some oil, which could have been digested to produce biogas.

Deshpande et al. (2012) studied the anaerobic degradation of mahua and hingan oil cakes [97]. Their investigation disclosed that both the seedcakes generated biogas in the scale of 198 to 233 l/kg of seedcake. They also found that both the sludge and the slurry have high fertiliser value. It is due to the presence of high nitrogen contents and other nutrients. Singh and Mandal (2011) studied various percentages of the non-edible oil cakes such as jatropha, karanja and safflower with cow dung for biogas production in a 1 L anaerobic batch reactor [98]. This experiment revealed that the array of the average yield of biogas was 0.236 to 0.363 L/g biogas 0.497 to 0.521 L/g VS after 41 days of digestion at 35 °C using several proportions. The corresponding methane content in the biogas was 2.5, 14.8 and 6.6% greater compared to cow dung only. It is easily surmised that each one of these non-edible oil cakes along with cow dung in 1:1 ratio would result in the best way of utilising these non-edible oil cakes. Hashemi et al. (2020) focused on the biorefinery prospect of safflower plant [99]. They digested the safflower seed cake in a 118 mL anaerobic glass bottle for 45 days. Their work concluded that 1 kg of safflower seed cake resulted in 146 L of methane. Gupta et al. (2012) assessed the use of both the raw and the detoxified cake for biogas production [100]. They reported a substantial enhancement in the biogas (93%). They also studied on mushroom yield, and the increase was 128%. Additionally, Gupta et al. (2013) also compared the biogas yield from raw and detoxified mahua seed cake [101]. They found that detoxified mahua seed cake delivered improved results contrasted to raw cake. It also reduced cellulose significantly to 34.4% and hemicellulose to 29.7%. The digested slurry had higher NPK contents. A similar study is also reported by Inamdar et al. (2015) [102].

Bateni and Karimi (2016) worked on another oil cake, *Eruca sativa* (Brassicaceae) [103]. It is an annual herbaceous plant dispersed in the Mediterranean region. They employed sodium hydroxide pre-treatment to improve biogas production from the plant residues. The study was conducted at two temperatures, 0 °C and 100 °C. Also, Bateni et al. (2017) studied the different oil extraction methods on biogas production [96]. The seed cakes were of two types, such as the mechanically extracted seed cake (MESC) and solvent extracted seed cake (SESC). Both the cakes were subjected to sodium hydroxide pre-treatment before anaerobic digestion. They noticed that pre-treatment considerably increased the methane production from the mechanically extracted seed cake by 105.6%, but returned deleterious effects on the solvent extracted seed cake. The methane production was approx. 210 mL/g VS for untreated cake. It increased to 410.6 mL/g for mechanically extracted seed cake but reduced to 140 mL/g VS for solvent extracted seed cake.

## Future recommendations and perspectives

The circular economy is at the heart of most of the SDGs. Its’ emphasis is rightly the optimal and sensible uses of resources, avoiding the wastage as much as possible. Biodiesel and biogas production are very much integral to circular economy ambitions. Despite all the trials and research reports, biodiesel production from vegetable oils continues to face many critical issues such as unutilised biomass generation. It results in the process being not so economically attractive, especially for large investments. In the absence of clear-cut economic benefits of biodiesel production, oil extraction from the oil seeds will remain questionable. In that case, there would be no oil cake [104]. If the biogas generation is coupled with biodiesel, the non-edible oilseeds provide a very viable economic investment. As many of the cakes are toxic apart from non-edible, they could be a substantial front for bioenergy generation with proper planning [100].

Additionally, waste to energy presents a vital alternative for the dual purpose of waste management and energy generation. Integration of anaerobic degradation of cake with biodiesel production is an exciting alternative for energy generation from non-edible oilseed cakes. A sustainable society will depend heavily on the circular economy, and biogas is the key to a circular economy [105–108].

The galloping consumption of energy in modern society necessitates an aggressive approach to renewable energy. The shortage of fossil fuels has led to the development of electric vehicles. Still, the electric vehicles are going to put stress on the supply of energy to the industrial and domestic households. The readily available biogas could easily compress these gaps. The uniqueness of biogas lies in its pristine characteristic of being generated from the waste. Researchers have discovered various wastes for generating biogas such as sewage, industrial wastewaters, municipal wastes and many other organic wastes. The waste oil cakes present another attractive option for the biogas generation. However, oil cakes to biogas centred circular economy involve a cohesive approach tackling waste management, utilisation and policy. Several factors need to be addressed for the development of a proficient oil cake to biogas based sustainable economy [99]. Since it is a blossoming technology, the supply chains are not identified. There is a lack of technical knowledge of efficient cake generation and corresponding economic value. In the absence of that, various stakeholders such as farmers, oil millers, traders and power plants will not be inspired to finance in this sector. Many countries in the world do not have well-defined policies and incentives to grow this sector. Besides that, various land protection laws and system hinder the promotion of biogas projects. The high population density in certain countries will also create an impediment for assigning the land for biogas projects. Other obstacles could be legal issues, grid connection issues, lack of subsidy, etc.; the development of Agri-based products such as dairy and farming is facilitated by a corresponding expansion of cooperatives and the associated organisations. Oil cake development will also need such cooperatives. The lack of a cooperative culture in many countries could be a serious hindrance in developing this sector [105].

Thus, the biogas production from oil cakes necessitates deeper study and more in-depth cooperation concerning techno-economic feasibility, policies and social acceptance. The supply chain must be clearly identified considering various aspects such as collection, storage and transportation. Furthermore, a great amount of training and awareness program should be conducted. Especially, the city planners and community developers should be encouraged to learn about this exciting sector. The pitfalls of dumping the oil cakes to the community, society and the country must be clearly expounded. A more significant discussion is necessary at the policy level highlighting various positives of non-edible oil cake to biogas route. As of now, the non-edible oil cakes are not looked upon favourably; the amount of biogas and bioenergy, which could be generated from these cakes, is far short of their potential. The proper training and policies will help bridge the gap.

Based on our work, the future in this sector should concentrate on the following factors:

The blending of fossil fuels with biodiesel from non-edible oils. Aggressive blending would generate huge oil cakes, which can be harnessed for biogas.

Availability of other Agri-wastes which can complement the biogas production from the oil cakes.

A detailed cost comparison of a large centralised plant with respect to small cooperative/private plants, especially in terms of public funding.

The application of biogas in the local context such as grid connection, cooking gas or other bioproducts generation.

Study on the application of anaerobic digestate as manure for the local soil types.

Detailed life cycle analysis of the process, considering the multitudes of ecosystem services.

## Conclusions

The future of the energy sector is going to be governed by renewable, carbon-neutral sources like biofuels which are far more sustainable than current dependence on fossil fuels. Biogas has been proven to be a reliable alternative gaseous biofuel that can be produced from a variety of organic substrates which are otherwise classified as waste. This paper endeavoured to carry out an exhaustive literature review on biogas generation from edible and non-edible oil cakes, a known Agri-waste. Critical analysis of the literature shows that oil cakes/meals produced as by-products during oil extraction from edible and non-edible seeds, by the virtue of its high nutritional content, could act as an excellent substrate for biogas production. As oil production is showing an upward trend in recent times, the expected output of oil cakes is also increasing. Biogas production from these oil cakes could be enhanced many folds by co-digestion with other substrates like animal manure. The edible oil cakes have been traditionally used mainly as animal fodder, but non-edible cakes do not have that utility. Therefore, they can be diverted for biogas production entirely. Among edible cakes, sunflower cake is widely studied in producing biogas, whereas jatropha oil cake is predominant amongst non-edible oil cakes. The review illustrates that the microbial inoculum is vital in anaerobic digestion for production of biogas as different sources have been shown to affect the yield. Newer studies are focusing on analysing the community composition by DNA sequencing and correlating it to the yield. This in-depth understanding about the interplay of different groups and community dynamics will go a long way in achieving a better outcome. Furthermore, the reactor design and keeping the process parameters in the desired range are being investigated to achieve better results. Various clean-up strategies are being used to remove impurities to increase the energy potential of biogas, and biogas upgrading is emerging a field in itself. The review mainly discusses different techniques for removing CO_2_ to improve the commercial applicability of biogas.

To achieve the goal of producing biogas from oil cakes in an economically favourable way, societal acceptance, robust supply chain and policy framework should go hand in hand with technological advancements. Few recommendations have been included in the previous section based on our survey of literature and keeping in mind the recent trends in the circular economy. The prospects and challenges discovered in this work could be a guiding light for exploring the biogas generation from these Agri-wastes.
